# Advantages and Disadvantages of Bioplastics Production from Starch and Lignocellulosic Components

**DOI:** 10.3390/polym13152484

**Published:** 2021-07-28

**Authors:** Mateus Manabu Abe, Júlia Ribeiro Martins, Paula Bertolino Sanvezzo, João Vitor Macedo, Marcia Cristina Branciforti, Peter Halley, Vagner Roberto Botaro, Michel Brienzo

**Affiliations:** 1 Institute for Research in Bioenergy (IPBEN), São Paulo State University (UNESP), Rio Claro 13500-230, SP, Brazil; mateusabe40@gmail.com (M.M.A.); julia.rima95@gmail.com (J.R.M.); joaovcmacedo@gmail.com (J.V.M.); 2 Department of Materials Engineering, São Carlos School of Engineering (EESC), University of São Paulo (USP), São Carlos 13566-590, SP, Brazil; paula.sanvezzo@usp.br (P.B.S.); marciacb@sc.usp.br (M.C.B.); 3 School of Chemical Engineering, The University of Queensland, Level 3, Don Nicklin Building (74), St Lucia, QLD 4072, Australia; p.halley@uq.edu.au; 4 Science and Technology Center for Sustainability—CCTS, Federal University of São Carlos, Rodovia João Leme dos Santos, Km 110, Sorocaba 18052-780, SP, Brazil; vagner@ufscar.br

**Keywords:** bioplastics, starch-based bioplastics, lignocellulosic fibers, extraction process

## Abstract

The accumulation of plastic wastes in different environments has become a topic of major concern over the past decades; therefore, technologies and strategies aimed at mitigating the environmental impacts of petroleum products have gained worldwide relevance. In this scenario, the production of bioplastics mainly from polysaccharides such as starch is a growing strategy and a field of intense research. The use of plasticizers, the preparation of blends, and the reinforcement of bioplastics with lignocellulosic components have shown promising and environmentally safe alternatives for overcoming the limitations of bioplastics, mainly due to the availability, biodegradability, and biocompatibility of such resources. This review addresses the production of bioplastics composed of polysaccharides from plant biomass and its advantages and disadvantages.

## 1. Introduction

Over the past two centuries, the significant growth of the world population and its consumption habits have led to several negative impacts on the environment. The development of a society with more sustainable production/consumption mechanisms should consider scenarios such as deforestation, water pollution, soil silting, and solid waste accumulation. Regarding plastic wastes, they represent approximately 12% of the composition of the world’s solid waste [1], and their annual production has been increasing since 1950 and exceeded 6 billion tons of waste generated between 1950–2015 [2].

Despite technologies and bioproducts (e.g., bioplastics) being an alternative for the mitigation of such environmental problems, a total replacement of synthetic plastics from petrochemical origin can hardly be considered in the short or even long term. On the other hand, certain applications of bioplastics may represent areas of large-scale potential replacement [3]; for example, biodegradable materials for packaging and other short-lived use sectors are viable, since they constitute a large part of the total plastics production [4,5,6,7].

Bioplastics can be classified into materials derived directly from natural polymers (agro-polymers), with or without modifications (e.g., starch-based bioplastics and/or cellulose), polymers produced by microbial fermentation (e.g., polyhydroxyalkanoates-PHAs), and biomaterials chemically synthesized from renewable raw materials (e.g., polylactic acid—PLA, bio-polyethylene-BPE, bio-nylons, and bio-polyurethanes). BPE is derived from the polymerization of ethylene from bio-ethanol, bio-nylons are produced via diacids from biomasses, and bio-polyurethanes are fabricated from the incorporation of polyols of plant origin [8]. However, even oils can represent a feedstock for the development of bioplastics.

Bioplastics from agro-polymers are derived from natural polymers such as polysaccharides (starch, cellulose, pectins, hemicellulose) and proteins (casein, zein, gluten, gelatin) that generally involve intra and intermolecular interactions and cross-links (crosslinking) between polymeric constituents, forming a semi-rigid three-dimensional polymeric network which retains the solvent [3,9].

However, large-scale production of bioplastics for different applications is limited by high costs, in comparison to synthetic plastics derived from fossil oil, and concerns over functionality [10]. Different biopolymers used have disadvantages such as high-water vapor permeability, oxygen permeability, fragility, low thermal resistance, low mechanical properties, vulnerability to degradation, and low processability [11,12,13,14,15,16,17,18,19].

The production and use of bioplastics instead of synthetic plastics (non-biodegradable and oil-based ones) reduce emissions of polluting gases and provide materials from renewable and/or biodegradable sources, availability of raw materials, and a promising alternative for the destination of solid biomass residues. Regarding environmental problems such as the greenhouse effect (the emission of greenhouse gases is a growing global concern, according to the Intergovernmental Panel on Climate Change (IPCC)), a 50% reduction in GHG emissions by 2050 is required for avoiding a 2 °C increase in the global temperature. Biomaterials such as bioplastics and biofuels are considered one of the mitigating measures in relation to global warming [5,7,20,21,22].

This review focuses on the different possibilities of bioplastics production from starch and lignocellulosic fibers, their advantages and disadvantages, and the procedures for obtaining natural polymers from plant biomass. Therefore, considering the development of several studies on the production of bioplastics from lignocellulosic fibers (from fractionation of the components or in nature and modified), in addition to the different applications that these bioplastics present, a review study on the characteristics (properties) of bioplastics and the processes for obtaining these polymers are justified.

## 2. Starch-Based Bioplastics

### 2.1. Characteristics and Structure of Starch Grain

**Figure 1:**
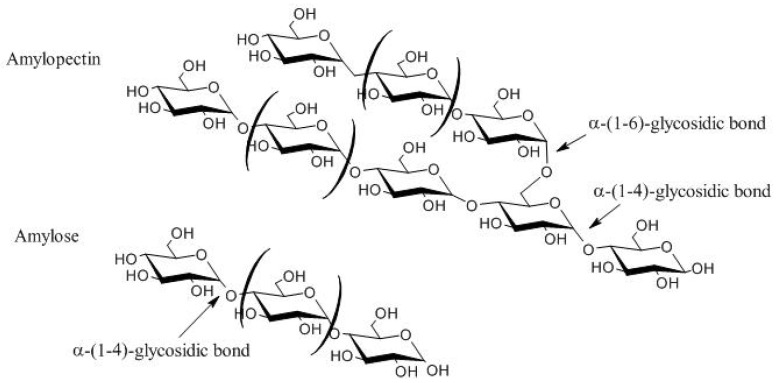
Representation of the starch structure of amylose and amylopectin.

Starch has a great industrial appeal due to different industrial sectors use it for applications such as beverages, textiles, paper, and pharmaceuticals [23]. Starch is insoluble in water and alcohol, composed of molecules of amylopectin and amylose, which are composed of monomers of d-glucose (Figure 1), and represent the main storage polysaccharide in the vegetal cells.

Similarly, to amylose and amylopectin, starch is a semi-crystalline polymer with linear regions; however, the α (1–6) branches of amylopectin reduce the degree of organization of the polymer [24,25]. Amylopectin consists of a short chain of 10–60 units of glucose in a linear and branched-chain, interconnected by α (1–4) and α (1–6) glycosidic bonds. Amylose is comprised of an unbranched linear chain of glucose monomers interconnected by α (1–4) glycosidic bonds. On average, the percentage of amylopectin and amylose contained in starch ranges between 72–75% and 25–28%, respectively [23,26].

### 2.2. Formation of Filmogenic Starch Solution

**Figure 2:**
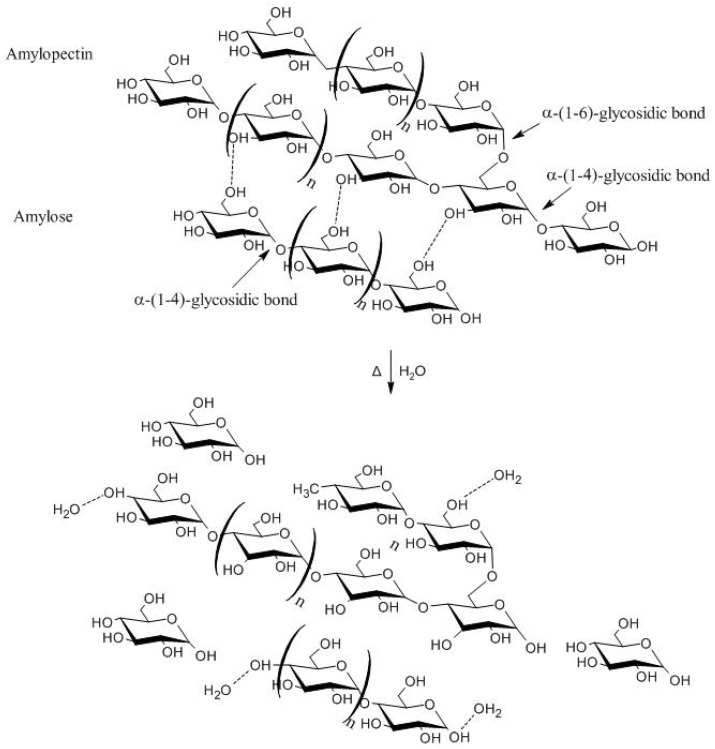
Gelatinization of starch.

Native starch molecules are linked through intermolecular interactions of the hydroxyl group and the oxygen of amylopectin and amylose. The amylopectin and amylose are joined by hydrogen bonds, which make it insoluble in cold water. However, the molecules can undergo significant changes when exposed to gelatinization temperatures, provoking the breakage of hydrogen bonds between starch components from the supply of thermal energy in an aqueous solution (Figure 2). In addition to the breakdown of hydrogen bonds, the viscosity of the starch solution increases between amylose and amylopectin, from amylose leaching, structural loss in the double helix of starch, birefringence, and interactions between water and starch through free hydroxyls [27,28,29]. Therefore, gelatinization begins in the amorphous regions of the polymer.

Another process that can occur mainly with amylose molecules is retrogradation, or starch retrogradation, according to which a gelatinized solution shows interactions between amylose molecules and an increase in the ordering degree of hydrogen bonds. Therefore, the amylose chains crystallize with the formation of a double helix [30,31], whereas starch retrogradation takes place more markedly when the solution is cooled. From the plasticizer effect of water and chemical plasticizers, starch gelatinization provides thermoplastic starch for bioplastics formation. In this process, starch loses its original conformation and forms a melted gel similar to synthetic thermoplastics, through swelling with water and other substances [32,33,34].

Bioplastics production from thermoplastic starch, or another natural polymer (polysaccharide, lipid, or protein), is completed with the deposition of the gelatinized filmogenic solution on a non-adherent surface. The solution is then dehydrated (in an oven for example), thus facilitating retrogradation due to the increase in intramolecular interactions between polymers from the reduction of the volume in the polymeric matrix. This procedure is known as Casting [35].

Apart from casting, other conventional methods of large-scale industrial production (e.g., extrusion and injection molding) are applied for bioplastics manufacture [11,12,13,14,15,16,17,18,19,20,21,22,23,24,25,26,27,28,29,30,31,32,33,34,35,36]. Single-screw and twin-screw extruders are the two main types used for polymer processing. The key advantages of the former are relatively low cost and a favorable performance/cost ratio, whereas the latter promotes a more complex velocity profile on the molten material, guaranteeing better distributive and dispersive mixing, heat transfer, and heat control. Besides, they both have flexible modular designs, and the screw configuration can be changed from soft melt mixing to vigorous mixing with high shear forces [37].

Regarding biocomposites, extrusion can be even more efficient, since both melting and mixing occur in a one-step process, which decreases polymer and reinforcement degradation, improves efficiency and suitability for industrial applications that require continuous processing [38,39]. However, the extrusion of biocomposites, particularly when nano-sized particles are used, can be challenging due to the dried nanomaterial’s tendency to aggregate, thus hampering the feeding of the material into the extruder. A possible approach for solving the problem is to feed the nanoparticles in liquid form (known as liquid-assisted extrusion) with the use of atmospheric and vacuum ventings along the extruder for the removal of vaporized solvents [40]. One of the most used technologies for processing polymers is injection moulding [41]; it provides a good quality/cost ratio when a large production is intended and can potentially process bioplastics and biocomposites [42,43].

**Figure 3:**
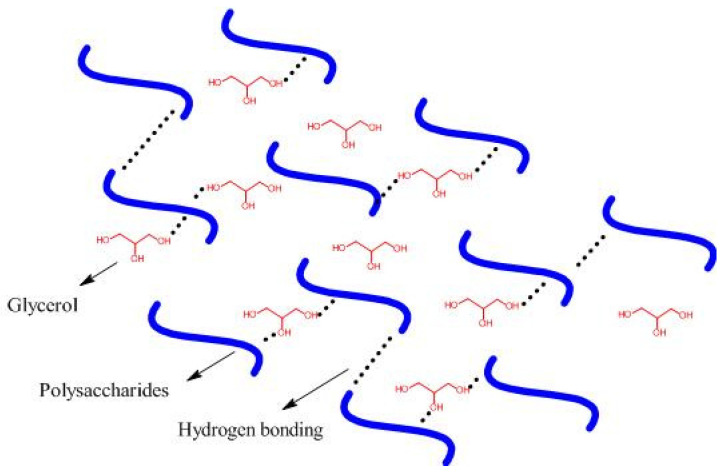
Polysaccharides and plasticizer interactions in the bioplastic matrix.

Polysaccharide-based bioplastics are brittle, non-continuous, rigid, and fragile when formulated with no additive [44,45,46,47] and plasticizers’ molecules used in the formulation of continuous bioplastics [48]. Examples of plasticizers are glycerol and sorbitol, which are compatible with polysaccharides. The plasticizer effect results in higher flexibility of the bioplastics due to an increase in the interstitial volume of the polymeric matrix [49]. Therefore, it reduces not only the glass transition temperature (*T*_g_) [45,46,47,48,49,50], but also the number of polymer-polymer interactions (Figure 3), and increases the molecular mobility and hydrophilic degree of the bioplastics [49,50].

The formulation of starch-based solutions with plasticizers increases water vapor permeability (WVP), elongation, and reduces tensile strength. Daudt et al. [50] reported the elasticity modulus and tensile strength in bioplastics of rice flour decreased with increasing glycerol concentrations, besides increased permeability to water vapor. The hydrophilic character of glycerol facilitates both adsorption and desorption of water molecules, thus increasing WVP [51].

Dias et al. [52] observed a reduction in the tensile strength of starch-based bioplastics from 10.9 MPa for 20% (*w/w* based on starch) of glycerol to 1.6 MPa for 30% (*w/w* based on starch). The same trend was observed with the replacement of glycerol for sorbitol as a plasticizer. The bioplastics with 20% and 30% of sorbitol showed, respectively, 22.3 and 11.2 MPa of tensile strength. Consequently, the reduction in the mechanical resistance increased elongation, and the use of 20% and 30% of glycerol resulted in 2.8% and 59.8% elongations, respectively. Bioplastics plasticized with glycerol showed lower resistance capacity and higher elongation than those plasticized with sorbitol at the same plasticizer concentration. The explanation for such differences lies in the smaller size of the glycerol chain, which promotes a higher plasticizer capacity in relation to sorbitol, i.e., glycerol shows a greater ability to interact with the matrix polymers and a larger amount of water is retained [53,54].

### 2.3. Properties and Characteristics of Starch-Based Bioplastics

Many of the properties and characteristics of starch are required for the use as coating or packaging material (e.g., biodegradability, biocompatibility, edible material (nutritional value), availability, relatively simple extraction process, and low cost) [55,56,57,58,59,60,61]. Other properties such as odorless, tasteless, and generally nontoxic, characterize starch as a molecule with the potential to be applied for packaging applications [62].

Other important features of starch-based bioplastics or any other polymer are color and transparency, related to marketing and consumer’s acceptance for a given product, depending on the bioplastics application. Table 1 shows some studies on starch-based bioplastics elaboration and properties.

Starch-based bioplastics have the disadvantages of hydrophilicity, poor mechanical properties, low water vapor barrier property, and low freeze stability during bioplastics formation [60,62,63,64,65]. However, depending on the amylose content in the polymeric starch matrix, certain bioplastics properties can change. Several procedures (e.g., physical, genetic, chemical, enzymatic, and others) are employed for altering the molecular structure of starch and, consequently, improving the properties of starch-based bioplastics [62,66].

The starch-based bioplastic properties are directly related to the raw material that originates starch [25], mainly due to the amylose and amylopectin proportion. This variation in starch molecule composition may be related to starch biosynthesis enzymes, soil type, and climatic conditions during plant growth [67]. Due to the difference in the amylose/amylopectin ratio of the different starch botanical sources, the gel and bioplastics produced can present different gelatinization temperatures, mechanical and rheological properties [67,68,69]. Thus, it is important to consider the type of starch used to elaborated bioplastics. For example, the starch-based bioplastic from yam presented tensile strength superior to those from corn and potato starch, due to the yam containing higher amylose content (29% *w/w*) [68]. Different gelatinization temperatures were reported (95.4, 92.5 e 88.5 °C) for several starch sources (potato, maize and waxy maize, respectively) (Hejna et al., 2019). Based on Daudt et al. [50] and Dias et al. [52], a comparison can be done using different starch sources and the same plasticizer concentration (glycerol 20%, *w/w*) in relation to the starch mass (Table 1). The studies reported the same elongation values, however, a remarkable difference in the tensile strength values. Therefore, these differences are due to the structure of the amylose and amylopectin chain, as a result of the more optimized hydrogen bonds between the amylose chains (not branched), which result in bioplastics less flexible and more rigid [68].

A low tensile strength and high elongation in starch arrowroot-based bioplastics, and improvement occurred in the tensile strength of starch-based bioplastics blending with gelatin [76] (Table 1). Bioplastics from this same source and with differences in the starch/gelatin ratio (with higher gelatin concentration) showed superior mechanical resistance [71] (Table 1). Gelatin protein is derived from the partial hydrolysis of collagen, and its properties are suitable for the formation of polymeric bioplastics. Gelatin increases intermolecular interactions due to an increase in the number of superficial protein chains [71,77,78].

The concentration and type of plasticizer can influence the bioplastics properties. Plasticizers increase the interstitial volume in the polymer matrix, which results in a reduction in the number of polymer-polymer bonds and thus affects mechanical [79,80], thermal [81], and barrier properties to water vapor [80,82]. Differences in mechanical and barrier properties of bioplastics can occur due to each plasticizing power. For example, due to the smaller size of the glycerol chain, it has a greater plasticizer character than sorbitol. Another feature of plasticizers is their hydrophilicity, which allows starch bioplastics to biodegrade faster than with sorbitol. Therefore, depending on the application and compatibility of the plasticizer with the polymer matrix, different plasticizers can be applied [83,84]. Bioplastics developed with *Renga pinnata* starch presented different properties using as plasticizers glycerol (G), sorbitol (S), and glycerol/sorbitol (GS) mixture (concentrations 15–45%, *w/w* starch basis) [79]. The tensile strength for bioplastics with G, S, and GS (15–45%) ranged between 9.59 to 1.67 MPa; 28.35 to 5.84 MPa, and 15.82 to 3.99 MPa, respectively. The yam starch-based bioplastic with different glycerol proportions (0–40% (*w/w*, starch basis) showed reduced tensile strength with increasing plasticizer content (49 to 10 MPa), in addition to increased flexibility (3 to 25%), and water vapor permeability (WVP) (6.75 to 7.59 × 10^−10^ g m^−1^ s^−1^ Pa^−1^) [68]. Other studies can be verified regarding the reduction of mechanical strength [81,85] and increase WTP with the addition of different hydrophilic plasticizers [85,86].

#### 2.3.1. Properties and Characteristics of Starch-Based Bioplastics Chemical Modified

**Figure 4:**
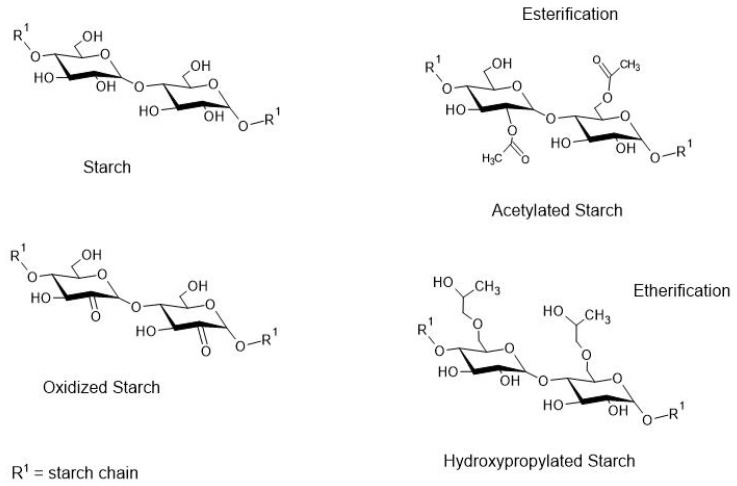
Representation of modified starch.

The chemical modification or the starch molecule derivatization may represent an approach aiming to improve the physicochemical and barrier properties of the native starch grain [87]. Some of the most common chemical derivatization reactions are esterification (acetylation), etherification (hydroxypropylation), and oxidation (Figure 4).

In the study by Abel et al. [88], the increase in the degree of acetylation and substitution of the starch hydroxyl groups (OH) led to a reduction in water absorption and water solubility. These results are consistent with the proposal of acetylation, in which the OH groups are replaced by acetic acid, thus reducing the hydrophilic character of the bioplastic.

Different degrees of acetylation and plasticizers can alter the mechanical properties, solubility, and water vapor barrier of starch-based bioplastics [89]. The degree of substitution (DS) of 0.6 was the most promising compared to the DS of 1.1. The DS of 0.6 resulted in a bioplastic with a tensile strength of 8.42 MPa, a solubility of 20.31 (g/100 g), and a WVP of 2.34 ×10^−7^ g.m/m^2^.h.Pa. While the tensile strength on the DS of 1.1 was 6.57 MPa, and the solubility and WVP did not change either. However, acetylation resulted in bioplastics with improved properties compared to native starch [89]. The improvement of bioplastics based on acetylated starch is due to the replacement of hydrophilic groups by acetyl, which results in a less flexible and hydroscopic bioplastic. Recent studies reported the effect of different acetylation on the starch-based bioplastic properties [90,91].

Starch oxidation can be performed with hypochlorite, hydrogen peroxide, permanganate, dichromate, persulfate, and chlorate are common. In this reaction the OH groups of glucose are replaced by carbonyl and carboxyl groups, usually on the carbon of number 2, 3 and or 6 [87]. Bioplastics elaborated with oxidized starch (dialdehyde) showed reduced water solubility (7.90–4.23%) and increased mechanical strength (1.63–3.06 MPa), from the increase in the degree of starch oxidation [92]. From different proportions of oxidized cassava starch (0, 20, 40, and 60%), the tensile strength (increased), flexibility (reduced), and water solubility (reduced) of the bioplastics were affected by the increase in the content of starch oxidation. However, above 20% of oxidized starch, there was no change in tensile strength [75].

By comparison, starch acetylation may result in a more hydrophobic and resistant bioplastic than oxidized starch-based bioplastics. This is due to the chemical nature of the hydroxyl substitution groups, that is, acetyl is more hydrophobic than carbonyl and carboxyl groups. Therefore, acetylated starch bioplastics hold less water and consequently result in a more compact matrix with optimized bonds between polymers. Acetylated starch bioplastics exhibited a contact angle of 60.41° and tensile strength of 16.35 MPa, while the same properties for the oxidized starch bioplastic were 45.47° and 13.38 MPa [91].

Hydroxypropylation in which the OH groups are replaced by hydroxypropyl ether results in the weakening of the interactions between the starch chains. Thus, as in the case of oxidation modification, starch modified with an ether group retains more water [87] when compared to acetylated starch. Bioplastics developed with a high percentage of amylose (75%) and 20% glycerol resulted in materials with increased flexibility and reduced tensile strength, from the increase in the propylene oxide content (6–12%, *w/w* starch). The tensile strength was 18.90 MPa (native starch), 15.66 MPa (6% propylene oxide), and 8.85 MPa (12% propylene oxide) [82]. Effects on mechanical properties (reduction) were also observed in starch-based bioplastic with propylene oxide [93].

#### 2.3.2. Chemical Starch Derivatization Impact on Biodegradation

Even though starch chemical modification in the manufacture of bioplastics can improve physicochemical and gas barrier properties, attention should be paid to the detriment of biodegradation properties [94]. Starch bioplastics can have the rate of biodegradation affected by chemical modification [92]. The reduction in the time and rate of biodegradation is related to the reduction in the degree of hydrophilicity of modified starch. From the chemical derivatization of starch, its solubility in water may be reduced, and in cases of optimization of the bonds between polymer chains, consequently, the interactions of the starch chain with water molecules are reduced. Organisms, especially microorganisms, need moisture to proliferate and metabolize the bioplastic.

Modified starch-based bioplastics showed a delay in anaerobic biodegradation, however, it is noteworthy that the degree of substitution of acetylated starch is >1.5, in which there is an impact on the biodegradation rate [95]. Bioplastic of PCL/acetylated starch blends composting degraded after 2 months 25.3% and 29.8% (80/20 and 60/40 PCL/acetylated starch, respectively) [96]. It is important to observe that the same proportion of PCL and native starch, disintegration was complete. Nevoralová et al. [90] demonstrated that the biodegradation of starch acetate-based bioplastics can be monitored by starch DS. In a composting system, starch-based bioplastics with high DS resulted in a lower mineralization rate than starches with low or moderate DS.

An alternative to improve the physicochemical and gas barrier properties of bioplastics, beyond ensuring biodegradation, is to join different chemical derivatizations. Advantage can be taken from the different potentials of each chemical modification. This approach is known as dual-modification [87], and can influence starch grain properties [97]. This approach is interesting mainly due to the different degrees of hydrophilicity of acetylation, oxidation, and etherification.

## 3. Lignocellulose and Biomass

Natural polymers from lignocellulose have been used for improving the mechanical properties (limited application) of bioplastics [98,99,100], due to their biodegradation characteristics and reinforcement provided [100]. Moreover, lignocellulose is an alternative to non-biodegradable synthetic fibers [100,101,102], and its benefits for the production of biomaterials include wide availability, renewable nature, low cost, and competitive specific mechanical properties [103,104].

Due to their potential application, components of lignocellulosic fibers from sugarcane bagasse, rice straw, flax, Kenaf, hemp, forest wood, and other sources have been widely used for reinforcement [98,103,105,106,107,108]. Components from biomass, such as cellulose (nano-scaled, lignin, and hemicellulose, are a strategic alternative to improve the bioplastics’ properties (barrier, mechanical resistance, thermal resistance, solubility). Moreover, renewable and biodegradable resources have been used for minimizing the problem of the accumulation and disposal of solid urban and agro-industrial wastes. Therefore, the generation of organic solid wastes enables their use for biotechnological purposes [109,110,111].

Examples of high availability of biological vegetal sources include generation of sugarcane bagasse (global and annual production of approximately 1.69 million tons—Brazil is responsible for 43%) [112], banana pseudo-stem (Brazilian production of 4 tons for each ton of fruit harvested approximately) [113,114], cassava starch (28.6 million tons worldwide production) [115], wheat bran (Brazilian production of approximately 2.6 thousand tons) [116], and food waste (1.6 billion tons) [117]. Non-synthetic organic residues, mainly from organic compounds, represent an environmental problem if not disposed of correctly, due to their chemical compositions [118,119,120].

Lignocellulosic fibers (lignin, hemicellulose, and cellulose) can potentially be applied for the production of biomaterials and biomolecules such as bioplastics, thus reducing dependence on oil [121]. Their use in biotechnological procedures can lead to an expansion of biorefineries [121] and the generation of jobs in rural areas [5,7,20].

A mixture that uses natural biopolymers represents a source with potential for different applications. Among the different natural polymers, lignocellulosic residues from plant biomass are a resource for the production and improvement of the bioplastic’s properties, such as mechanical properties, in addition to the great availability of these fibers represent a possibility of cheaper bioplastics (in terms of raw material availability) and an alternative for the lignocellulosic waste management. Bioplastics produced from mixtures of more than one type of polymer can show improved properties in comparison to individual polymers [66].

### 3.1. Characteristics of Cellulose

Cellulose is the most abundant natural organic compound on the planet, with an annual production of approximately 180 billion tons [10,122]. It represents the main polysaccharide in the constitution of the plant cell wall, divided into primary and secondary walls. The latter is subdivided into three layers, namely S1, S2, and S3, of which S2 guarantees the resistance characteristic of vegetable cells due to the greater thickening (approximately 90% of the cellulose in micro and macrofibril forms) [123].

The characteristics of cellulose are insolubility in water, high molar mass, and arrangement in nanofibers. Cellulose displays regions of poor organization and others highly crystalline, characterizing a semi-crystalline fiber. Due to such characteristics and the high resistance to enzymatic hydrolysis (recalcitrance) [124], lignocellulosic fibers provide plants with a structure that resists environmental and biological weathering [125].

**Figure 5:**
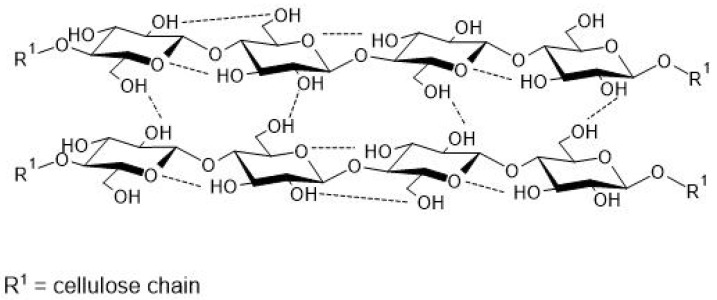
Partial structure of cellulose.

Cellulose is biosynthesized not only by plants, but also by microorganisms such as algae, fungi, and bacteria [126,127,128]. Regardless of the organism or cellulose synthesis pathway, this polymer is classified as a homopolysaccharide of high molar mass and beta glycosidic bonds (1–4) [127,128,129]. Its degree of polymerization (DP) is higher than 10,000 units of anhydroglucose; however, it varies according to the botanical source [129]. The properties of cellulose such as mechanical resistance and reduced interaction with water molecules are due to its arrangement. Polysaccharide chains that interact through hydrogen and hydrophobic bonds, via glucose monomers, resulting in a conformation of planar sheets [129]. Figure 5 displays the structure and interaction of cellulose chains. The glycosidic bond between two glucose monomers in the same cellulose polymer results in cellobiose [128,129].

Therefore, cellulose can potentially be used for the manufacture of biomaterials such as bioplastics, and in several areas and industries (e.g., cosmetic, food, and pharmaceutical industries [130,131] due to its biodegradability, availability, non-toxicity, and biocompatibility [132].

### 3.2. Bioplastics with Cellulose

Cellulose is biodegradable and renewable [133], which is advantageous for sustainable applications. Improvements in the properties of different bioplastics, such as those based on starch and hemicellulose, with the addition of cellulose are one of its main advantages. The addition of cellulose and carboxymethylcellulose to starch-based bioplastics reduced the water vapor permeability (WVP) [35,56]. However, the mechanical resistance increased from 3.9 to 9.8 MPa, and elongation at break was reduced from 42.2 to 25.8% [19].

In comparison with pure thermoplastic starch, WVP is lower in cellulose bioplastics, however, starch-based bioplastics with cellulose showed lower tensile strength values than pure starch-based bioplastics [12]. The mechanical properties of starch-based bioplastics with cellulose can be reduced with the addition of macro-size cellulose, due to the formation of energy concentration points in the bioplastic matrix when subjected to an axial force. Furthermore, the use of cellulose fibers on a macro scale may not represent an adequate reinforcement, since fibers agglomerate in the polymer matrix due to the different microfibers sizes. This result was reported by [134,135], who used fresh pea husk fibers and fibers processed by acid hydrolysis, and by [136]. Therefore, the processing of cellulose alters the bioplastics’ shape and structure, hence, their properties [136].

The reduction and standardization of cellulose fiber dimensions favor its application as a reinforcement for bio-nanocomposites. Nanofibers smaller than the macro scale are classified as nanoparticles (three dimensions on the nanoscale), nanotubes (two dimensions on the nanometric scale and a larger one (the third), forming an elongated structure, referred to as nanowhiskers), and nanolayers (only one dimension on the nanometric scale, in a sheet form) [137].

The literature reports improvements in the mechanical properties of biomaterials with the addition of cellulose nanowhiskers [72,134,135,138,139,140]. Gordobil et al. [141], and Hansen et al. [142] also observed an increase in mechanical strength with the use of hemicellulose-based bioplastics with nanocellulose. Such improvements are achieved through an adequate dispersion of the nanofibers in the polymeric matrix and optimization of the hydrogen bonds in the bioplastic matrix. The interaction between polysaccharides and cellulose as reinforcement is favored by chemical similarity and intermolecular hydrogen bonds between hydroxyl groups of macromolecules [135,138,143]. In addition to mechanical reinforcement, biocomposites formulated with nanofibers have increased resistance to moisture due to the greater number of hydrophobic compounds [144]. Moreover, the strong intermolecular interactions between cellulose and matrix, which increase the tortuous path, increase crystallinity, glass transition temperature [145], and reduces the diffusion of water vapor through the material [145,146,147,148,149].

The amount of cellulose must also be considered in the production of cellulose-based bioplastics since its excess can cause agglomerations of cellulose granules in the polymeric matrix [134,135,144], thus reducing mechanical strength and barrier properties due to a lower optimization of hydrogen bonds [150,151].

#### 3.2.1. Properties and Characteristics of Cellulose-Based Bioplastics Chemical Modified

**Figure 6:**
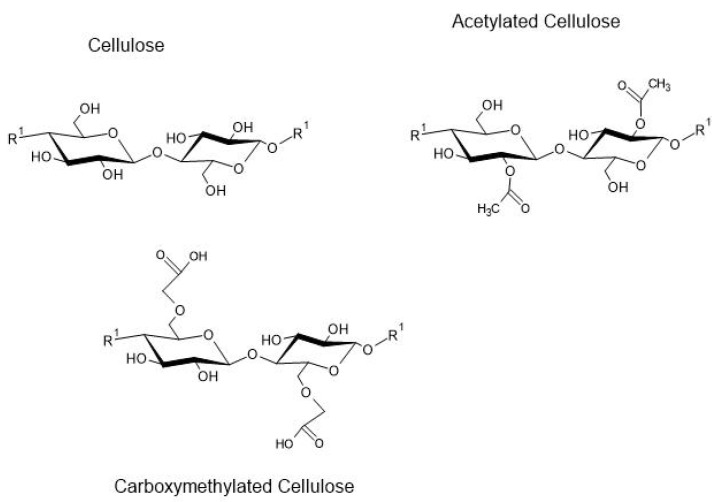
Representation of modified cellulose.

The main cellulose derivative produced from the etherification process (cellulose ether) is carboxymethylcellulose “CMC” [152,153]. The replacement of the hydroxyls of the cellulose monomeric unit by carboxymethyl groups occurs at carbon in the position 2, 3, or 6 (Figure 6). The use of CMC in the production of biomaterials is interesting due to its degradability, nontoxicity, and availability of raw material.

Regarding microcrystalline cellulose “MC”, certain characteristics of CMC for the bioplastics development are solubility in water at different temperatures (use of water as a solvent), and reduction in the formation of particle agglomerates with other polymers [154,155]. The hygroscopic character of CMC is dependent on DS, degree of polymerization, and distribution of ether group substitutions [155].

Starch-based bioplastics with added MC and CMC resulted in bioplastics with improved tensile strength (up to 9 wt%) compared to pure starch bioplastic [154]. However, bioplastic with MC resulted in a lower WVP and possibly less interaction with the starch matrix (due to a greater degree of apparent agglomeration (12 wt% of MC)). This higher WVP of the starch/CMC bioplastic may be due to the hydrophilicity of the carboxymethyl group in the CMC. A greater interaction with water and smoother surface morphology of bioplastics with CMC was reported [156]. The improvement of mechanical properties and increase WVP in bioplastics developed with CMC have been confirmed [153,157,158]. However, conflicting results [156,157] of the effect of CMC on solubility in starch-based bioplastics may be the result of different factors such as botanical source, crystallinity, purity, processing, and DS.

Cellulose acetate or acetylated cellulose (CA) (Figure 6), represents the native cellulose that has gone through the acetylation process. CA can be categorized into mono, di, or triacetate, with CA being insoluble in water. Cellulose DS is related to the reduction of WVP and the interaction of the bioplastic with water [159]. The reduction of WVP by the acetylation of cellulose is due to the replacement of hydroxyl groups (hydrophilic) by acetyl groups (hydrophobic) [160]. That is, inhibiting the accumulation of water in the bioplastic [161].

Xylan-based bioplastics showed reduced solubility in water and food simulants (30.88% to 16.30%) after increasing the DS of nanocellulose (0 to 2.34) [159]. In the same study, hydrophobicity increased from 24.59° to 62.68° with DS from 0 to 2.34, respectively. A reduction in tensile strength (3.62 MPa to 2.06 MPa) of acetylated starch and cellulose-based bioplastic occurred by the reduction of CA in relation to acetylated starch (SA) (1:9 and 7: 3 SA: CA, respectively) [160].

#### 3.2.2. Impact of Chemical Cellulose Derivatization on Biodegradation

The greater degree of hydrophobicity can result in a delay in the rate of biodegradation due to the reduction in the interaction of acetylated starch with water. The modification of cellulose may result in the need for different enzymes for hydrolysis, such as esterases, which are common enzymes for native xylan hydrolysis (native xylan contains acetyl groups) [162]. Therefore, the need for microorganisms that perform the deacetylation and de-esterification pathway is needed [163].

Increasing the concentration of acetylated starch, with a consequent reduction in CA, occurred a higher rate of biodegradation [160]. The pure CA bioplastic degraded 30% approximately in 120 days. However, with the lowest CA concentration in the starch-based bioplastic, 52.43% biodegradation occurred after 120 days. Acetylated cellulose-based bioplastics showed a delay in anaerobic biodegradation, however, it is noteworthy that the degree of substitution of CA was >1.5, in which there is an impact on the biodegradation rate [95].

From landfill 35 bacteria were isolated showing growth halo in CA emulsifying medium, however, few isolates degraded CA bioplastic with DS 1.7 [164]. The strain S2055 (*Bacillus* sp) was the only isolate capable of degrading more than 10% of the CA bioplastic. It is known that CA with a high degree of substitution is more resistant to biodegradation [165,166]. For more information, see the indicated specialized literature about the biodegradation of CA by thermophilic aerobic and anaerobic microorganisms [167,168].

The hydrophilic character of the carboxymethyl groups on the surface of CMC represents an important aggravating factor in maintaining bioplastic integrity [169]. The greater affinity of CMC with water in relation to CA can affect the biodegradation of carboxymethyl cellulose-based bioplastics. This effect of stimulating biodegradation due to the swelling of CMC with water may be related to the role of pH. A possible explanation for an acceleration of polymer degradation (abiotic and/or enzymatic) is the process of deprotonation of carboxylic acids and the formation of carboxylate groups, which have a high affinity with water [170]. Above pH 4.6, CMC is in its deprotonated form [171].

A proof that CMC is highly biodegradable, susceptible to microbial enzymes, and soluble in water, is its use in methods to verify the cellulolytic potential of different microorganisms. In these methods, microorganisms are cultivated in media with CMC as the only carbon source, and the growth halo is indicative of the production and secretion of cellulolytic enzymes.

### 3.3. Hemicellulose

**Figure 7:**
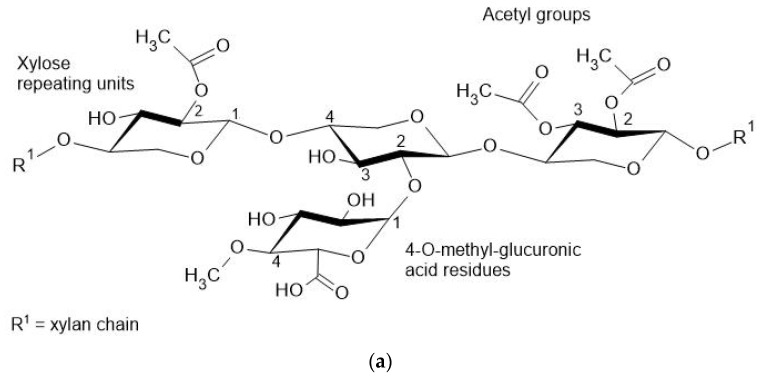

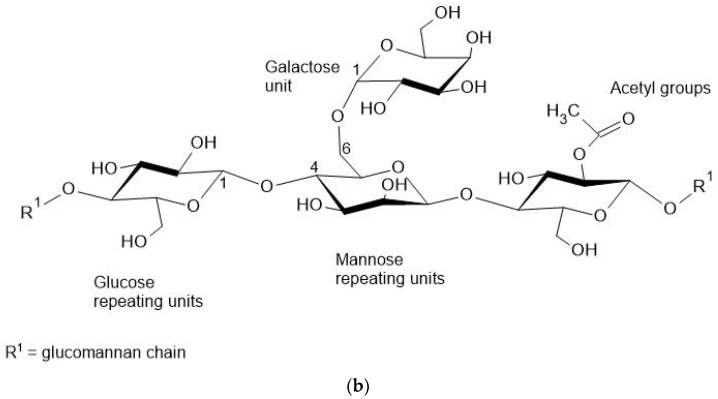
Representation of hemicellulose constituents(**a**) O-acetyl-4-O-methylglucoronoxylan, (**b**) O–acetylgalactoglucomannan.

Hemicellulose is a constituent of plant biomass; together with lignin and cellulose, they become the major constituents of the plant cell wall, forming a lignocellulosic complex. In this complex, it represents the second most abundant polymer in lignocelluloses [172,173,174]. However, unlike cellulose, it consists of different monomeric units, namely mannose, arabinose, xylose, glucose, and galacturonic acid [121,175], whose content can vary according to the botanical origin [121]. Figure 7 illustrates such constitutional differences. In hardwoods, the major component of hemicellulose is O–acetyl–4–O–methylglucuronoxylan (with substitution on carbon 2 of xylopyranose backbone units by 4-Omethyl glucuronic acid and 70% substitution on carbon 2 and 3 of the xylopyranose units by acetyl group). In softwood, however, the main hemicellulose component is O–acetylgalactoglucomannan, whose structure is formed mainly by units of glucose and mannose through beta (1–4) interactions with alpha (1–6) linked galactose units attached to the glucose and mannose units [121]. The acetylation degree in carbon 2 and 3 corresponds to approximately 20% [176,177].

Xylan is one of the constituents of hemicellulose present in different agroindustrial residues (sugarcane bagasse, wheat straw, sorghum, corn stalks, and cobs), besides forest residues and hardwood pulping [124,178,179]. In other words, xylan is the monomeric carbohydrate predominate in most plants, in addition to representing one-third of the renewable biomass on planet earth [178]. All these characteristics make it a potential source of biotechnological applications such as bioactive compounds and the manufacture of biomaterials.

#### Bioplastics with Xylan

Since hemicellulose is highly available, it is a promising source for obtaining chemicals and materials [174,175,180,181], however, its applications are limited due to its high heterogeneity, low mechanical properties, hydrophilic character, and difficult formation of continuous bioplastics [45,182,183]. Hemicellulose can be used in bioplastics manufacture, medical applications, hydrogels, and cosmetics [184], due to its biodegradability, biocompatibility, and easy chemical changes [185,186]. However, it requires alterations for providing better properties and, consequently, achieving higher valorization. Chemical changes in hemicellulose and crosslinks between hemicellulose molecules and blends with other polymers are alternatives for overcoming the difficulties in its use.

Citric acid was used as a crosslinking agent in xylan-based bioplastics blend with polyvinyl alcohol [49]. A strong link between the filmogenic matrices was observed due to the formation of ester bonds between the molecules and hydrogen bonds. The bioplastic elongation increased from 15.1% to 249.5%; however, the tensile strength was reduced when citric acid acted as a plasticizer.

Gordobil et al. [141] developed filmogenic solutions with modified xylan and nanocellulose to improve the bioplastics’ physical properties. The acetylation and bleaching of the polymeric components increased both tensile strength and Young’s modulus and reduced elongation. Gordobil et al. [141] and Stevanic et al. [187] demonstrated the addition of adequate amounts of nanocellulose to xylan-based bioplastics increased the bioplastics’ mechanical properties and hydrophobicity. The authors obtained higher values than those achieved by Goksu et al. [45], who used the only xylan in the formulation of the bioplastic matrix and reported lignin is necessary for the obtaining of continuous xylan-based bioplastics.

The blending of hemicellulose with other polymers can improve the properties of biomaterials formed by hemicellulose. Several studies have reported the addition of biopolymers to hemicellulose-based bioplastics as a strategy to improve their mechanical properties. Table 2 shows the mechanical properties of blends of hemicellulose-based bioplastics with biopolymers. After an extensive review of the literature, the authors of this review did not find studies that reported the development of blends of bioplastics with starch and xylan, therefore, the present work addresses a new research strategy on bioplastics.

As shown, different proportions of nanocellulose and plant raw materials of hemicellulose result in different tensile strength values, elongation, and elasticity or young modulus. According to the tensile strength data in Table 2, the resistance values of hemicellulose-based bioplastics reinforced with biopolymers (as micro and nanocellulose) are close or even higher than those of commercial non-biobased polymers (e.g., Cellophane^TM^ and low-density polyethylene (LDPE)) [142,192,193]. However, elasticity is one of the properties that require improvements [142].

The reasons for using cellulose as reinforcement (Table 2) are due to its chemical similarity and compatibility with hemicellulose [142], as well as the strong interactions between cellulose and hemicelluloses of xyloglucan type and between cellulose and glucomannan [142,194,195,196]. Moreover, cellulose nanofibers improve bioplastics, reducing their rate and permeability to water vapor. Hansen et al. [142] and Saxena and Ragauskas [196] showed that nanocellulose addition improves the properties of xylan-based bioplastic. Due to its high crystallinity (higher than 60%) and dense network of polymeric bonds formed by hydrogen bonds, whisker-type nanocellulose produces a tortuous path in bioplastics, since it works as a barrier structure, thus hampering the transport of water molecules through the material [196].

As in the case of starch and cellulose chain chemical changes, there are implications for the biodegradation of bioplastics based on modified hemicellulose, however, the information in the literature is limited. Therefore, it is highly recommended to evaluate the influence of polysaccharide modifications on biodegradation [94].

## 4. Extraction of Starch and Lignocellulosic Components (a Challenge)

Regarding the development of bioplastic materials with the addition of natural polymers such as lignocellulosic fibers, the steps that precede the formulation and modification stages of such materials must be considered, since the extraction and purification processes of the lignocellulosic fibers from the biomass are responsible for the economic viability of the fiber’s application, hence, the final material’s price. The processes will also define the quality and characteristics of the final materials because they influence the properties of the bioplastic films formed and their suitability for a particular application.

The choice of biomass must be considered for manufacturing processes and further transformations of starch, due to its different characteristics and compositions. Tubers, for example, contain a very small amount of proteins and fats, which facilitates the isolation of starch [197]. The most common sources of commercial starch are cereals such as corn, rice, and wheat, with more than 60% starch, as well as the roots or tuberous of cassava and potato, with approximately 16–24% of starch in weight [198,199,200].

The unit operations for the extraction of starch start with disintegration—the plant cell walls are opened, thus exposing their starch granules [201]. The next steps are extraction, in which starch is separated from the fibers, and purification, especially from proteins. Starch is then concentrated and finally dried [202].

The industrial process of starch isolation separates starch from protein, usually using an alkaline solution [203,204]. Different alkaline agents, such as detergents and sodium hydroxide or sodium hypochlorite, can be employed as extraction solvents [205]; however, concerns over the disposal of effluents arise due to their use [203]. Such methods have been proven effective for the production of starch films [206], and important considerations include avoidance of amylolytic or mechanical damage to the starch granules, effective deproteinization of starch, and minimization of the loss of small granules [207].

Hydrothermal processing with microwave-assisted extraction can optimize the process, since it is considered a green and safe technology for starch extraction, due to its ease of use, the possibility of using only water as extraction solvent, short extraction times, higher performance, and lower solvent consumption [208,209]. Therefore, it has been applied on an industrial scale for the obtaining of bioactive compounds [210].

In cost-effective and efficient isolation of cellulose from biomass, the cellulose source should ideally come from economically viable and easily accessible agro-wastes, as the amount of cellulose in various natural sources can vary, depending on the species and the lifetime of the plants. From a technological point of view, the evaluation of lignin content is crucial for the optimization of the pretreatment necessary for the extraction of pure cellulose pulp [211]. Indeed, lignin is considered the hardest chemical component to be removed from lignocellulosic materials [212].

Initially, the material is subjected to a water-washing process for the removal of dirt/impurities and water-soluble extractives. The biomass compounds closely linked to cellulose, such as hemicellulose and lignin, are then removed. The complexity of the composition of lignocellulosic materials hampers the penetration of chemical agents, thus requiring a pretreatment for the breakage of the structure and facilitation of chemical processes, hence, economy.

Kraft pulping uses a mixture of sodium hydroxide (NaOH) and sodium sulfide (Na_2_S) in a digester to dissolve lignin and hemicellulose [211]. The strong base disrupts OH bonding in the fiber network structure by ionizing the hydroxyl groups of various materials in fibers [213]. Such a process is addressed in research on cellulose extraction for film formation and is widely used on an industrial scale, with 96% market dominance [211,214,215].

The addition of sodium sulfide facilitates ether cleavage and controls undesirable condensation reactions, resulting in a high yield of strong fibres. However, it generates sulfite derivatives, which may link to cellulose and cause environmental problems within disposal [216,217]. Many treatments free from chlorine and/or sulfide have been developed towards reducing the environmental impacts of the pulping process [211,218]. Due to strict environmental regulations, organosolv has emerged as an alternative owing to its unique features [216,217].

After pulping, the resulting material can undergo a bleaching step, or delignification, which uses different bleaching agents such as chlorine dioxide (ClO_2_), hydrogen peroxide (H_2_O_2_), ozone (O_3_), or peracetic acid [211]. The use of chlorine dioxide has excelled that of elemental chlorine in controlling parameters such as chemical and biochemical demand for oxygen and total solids, as it more effectively minimizes the polluting load of bleaching effluents. Significant pollution reductions have been achieved; however, its use still causes environmental concerns [219].

Similar procedures can be adopted for hemicellulose extraction, especially regarding bioplastics formation [159]. Since hemicelluloses exhibit an amorphous structure, they are more vulnerable to degradation than cellulose, and some extreme methods can be responsible for their hydrolysis into monomers. Although the alkali treatment under moderate conditions cannot break glycosidic bonds between hemicellulose monomers, it is suitable for obtaining hemicellulose of high polymerization degree [220].

The most applied hemicellulose isolation method involves an alkaline reaction usually with NaOH or KOH [159], which dissolves hemicelluloses and lignin, cleaving the phenyl glycoside bonds, esters, and benzyl ethers linkages between such structures, hydrolyzing uronic and acetic esters, and swelling cellulose, decreasing its crystallinity [221,222].

Low-boiling-point organic solvents such as ethanol, methanol, butanol, and acetone can be used in alkaline reactions for biomass fractionation for avoiding extremely high temperatures and reducing environmental impacts and energy consumption [223,224]. Although the method also recovers solvent by distillation, high costs are associated with wastewater used for washing the resulting material, which limits its economic viability on a large scale [225].

From an environmental point of view, enzymatic extraction is more acceptable than chemical procedures [226]. It uses specific hemicellulose-degrading enzymes to obtain hemicellulose from biomass, and, although slower than other methods, the degree of polymerization obtained can be controlled by both reaction time and enzyme activity applied.

The complete use of the biomass compounds is required so that the process becomes a more profitable investment. Therefore, the isolation of lignin with few changes in its structure may be advantageous, since it can be used for specific applications, such as the production of resins, adhesives, carbon fiber, activated carbon, among others [227].

During biomass fragmentation by alkali treatment, lignin is degraded into soluble fragments and then separated either with the removal of the reaction solvent or by lignin precipitation [228,229]. However, overly severe extraction conditions may induce substantial changes in the original lignin structure [229,230]. Among such processes, organosolv pretreatment with ethanol or acetic acid has been widely used [231], and organic acids such as acetic acid and formic acid yield a high-quality product [232].

## 5. Environmental Impact of Polysaccharide-Based Bioplastics from Plant Biomass

The following information provides an overview of the bioplastics developing implications, in order to complete the pros and cons of using plant biomass. This is an attempt to allow a broader study of the impacts of plant biomass-based bioplastics, without any intention to overshadow the clear importance and benefits of the biomaterials development and use.

The carbon footprint refers to the measurement (CO_2_ equivalent) of emissions of CO_2_ and other gases in the GHG (greenhouse gases) category [233]. The human need for natural resources of the biosphere for different services and products can be measured by the ecological footprint, and the water footprint refers to direct and indirect consumption demand for freshwater in the development of a product or technology.

In the study by Korol et al. [233] the carbon, ecological, and water footprints of cotton fibers (CF), jute (FJ), and kenaf (FK) added to synthetic plastic polypropylene (PP) were analyzed. The results showed, in relation to the carbon footprint, the CF, FJ, and FK fibers had a lower impact (3%, 18%, and 18%, respectively) compared to PP. This measurement is related to the use of energy and petroleum processing in the manufacture of propylene and polymerization. Regarding the ecological footprint, the FJ and FK fibers showed less impact (8.2% and 9.4% reduction, respectively), however, due to the cultivation and harvest of CF fibers occurring in greater quantity and not being manual (use of machinery and energy expenditure), these had a high ecological footprint (an increase of 52%). However, the water footprint in the study by Korol et al. [233], proved to be alarmingly more worrisome from the point of view of natural plant-based resource use. The use of fibers added to the PP pellet is responsible for 286% (FK), 758% (FJ), and 891% (CF) of the increase in the water footprint. The increase in the water footprint of plant biomass in applications of blends with synthetic polymers is mostly related to water resources applied in irrigation. Korol et al. [234] also observed the increase in the water footprint, resulting from the use of plant biomass, in another study.

The application of native starch in bio-plastics resulted in reduced GHG emission (up to 80%) and nonrenewable energy use NREU (up to 60%) [235]. These natural polysaccharides can result in an increase in the potential for eutrophication (up to 400%) and land use (0.3–1.3 m^2^ yr/kg), compared to petrochemical plastics. Moreover, these negative impacts about the use of bioplastics or additives based on plant biomass are debatable, as the implications of the arable land use and water resources due to the cultivation and harvesting of these biomasses can be mitigated through the approach of reusing agro-industrial and urban wastes. In addition to reducing the environmental impacts mentioned above, the use of waste from the wood industry, crops, and urban is a management alternative to agro-industrial and urban organic solid waste.

The life cycle assessment approach (LCA), blends with starch residues (waste from fries potato processing) depicted a reduction in the eutrophication potential (up to 40%), land use (up to 60%), GHG and NREU (reduction < 10% for both), compared to virgin starch [235]. The reduction of the water footprint can also be reduced through the use of residues from vegetal biomass, to take advantage of residues from different crops.

## 6. Conclusions

This review has addressed the state-of-the-art of the production of bioplastics from polysaccharides from plant biomass, as well as the advantages and disadvantages of using starch and lignocellulosic components (as an additive and main component) for their development. Academic and industrial efforts have been devoted towards new and improved polymers, production methods, and sources for the obtaining of polysaccharides that can strategically reduce petroleum consumption in the production of plastic and replace partially the conventional synthetic and non-biodegradable plastic materials. Moreover, the production of bioplastics from plant biomass represents a model for the recycling and management of such waste with positive economic effects. However, the disadvantages (mechanical resistance, gas barrier properties, processability of natural polymers, and economic viability) related to the production of bioplastics from polysaccharides must be studied towards the expansion of the fields of application of such materials. This study showed that the application of lignocellulosic fibers has a high potential for application in bioplastics, since they result in the improvement of the properties of bioplastics, in addition to being an alternative to reuse biomass with great availability.

## Figures and Tables

**Table 1:** Production and properties of starch-based bioplastics.

Starch Source (%, *w/v*)	Tensile Strength (MPa)	Elongation at Break (%)	Water Solubility (%)	Mixture * (%, *w/w*)	Plasticizer * (%, *w/w*)	Bioplastic Processing Method	Reference
Sweetpotato (2.5%)	7.96	77.92	…	None	Sorbitol (40%)	Casting	[70]
Manioc (3%)	64.29	3.87	20.81	Gelatin (25%)	Glycerol (10%)	Casting	[71]
Manioc (3%)	108.28	6.57	28.88	Gelatin (75%)	Glycerol (10%)	Casting	[71]
Corn (5%)	26	3.6	…	Cellulose nanocrystals (13%)	Glycerol (26%)	Casting	[72]
Corn (5%)	10	33.1	…	None	Glycerol (30%)	Casting	[72]
Reag (2%)	5.21	22.25	77.54	Papaya (80%)	Glycerol (30%)	Casting	[73]
Rice (5%)	10.9	2.8	…	None	Glycerol (20%)	Casting	[52]
Sugar palm starch (8%)	7.74	46.66	>31	None	Glycerol/Sorbitol 1:1 (30%)	Casting	[74]
Cassava (5.26%)	1.14	0.22	13.48	None	Glycerol (20%)	Casting	[75]
Pinhão (5%)	18.56	2.8	…	None	Glycerol (20%)	Casting	[50]
Arrowroot (2%)	3.9	45.3	…	None	Glycerol (30%)	Casting	[76]
Arrowroot (2%)	11.5	44.4	…	Gelatin (50%)	Glycerol (30%)	Casting	[76]

… = not reported, * = based on total dry mass.

**Table 2:** Properties of hemicellulose-based bioplastics.

Hemicellulose (% *w/w*) *	Mixture (% *w/w*) *	Tensile Strength (MPa)	Elongationat Break (%)	E (MPa)	Reference
Xylan (87%)	Nanocellulose (13%)	20.2	2.6	1578	[47]
Xylan (50%)	Nanocellulose (50%)	57	1.7	5700	[142]
Arabinoxylan (85%)	Microcellulose (15%)	95	<15	2500	[188]
Galactoglucomannans (85%)	Microcellulose (15%)	15–20	3–4	800–1000	[189]
Xylan (20%)	Gluten (80%)	7–8	1–50	130–150	[178]
Xylan (10%)	Microcellulose (90.9%)	160–175	…	160–175	[190]
Xylan (95%)	Nanocellulose (5%)	51	2.9	3200	[141]
Arabinoxylan (95%)	Bacterial Cellulose (5%)	68	8.1	2700	[191]
Arabinoxylan (75%)	Nanocellulose (25%)	108	6	4800	[187]

… = not reported. * = basedon total dry polysaccharides.
